# Robust 3D Face Reconstruction Using One/Two Facial Images

**DOI:** 10.3390/jimaging7090169

**Published:** 2021-08-30

**Authors:** Ola Lium, Yong Bin Kwon, Antonios Danelakis, Theoharis Theoharis

**Affiliations:** 1 System Development, Dfind Consulting, Akersgata 7, 0158 Oslo, Norway; olalium@gmail.com; 2 Department of Computer Science, Norwegian University of Science and Technology, Gløshaugen, Sem Sælands vei 9, 7034 Trondheim, Norway; yongbinkwon97@gmail.com (Y.B.K.); theotheo@ntnu.no (T.T.)

**Keywords:** 3D face reconstruction, rotated face generation, 3D face analysis, convolutional neural network, computer vision

## Abstract

Being able to robustly reconstruct 3D faces from 2D images is a topic of pivotal importance for a variety of computer vision branches, such as face analysis and face recognition, whose applications are steadily growing. Unlike 2D facial images, 3D facial data are less affected by lighting conditions and pose. Recent advances in the computer vision field have enabled the use of convolutional neural networks (CNNs) for the production of 3D facial reconstructions from 2D facial images. This paper proposes a novel CNN-based method which targets 3D facial reconstruction from two facial images, one in front and one from the side, as are often available to law enforcement agencies (LEAs). The proposed CNN was trained on both synthetic and real facial data. We show that the proposed network was able to predict 3D faces in the MICC Florence dataset with greater accuracy than the current state-of-the-art. Moreover, a scheme for using the proposed network in cases where only one facial image is available is also presented. This is achieved by introducing an additional network whose task is to generate a rotated version of the original image, which in conjunction with the original facial image, make up the image pair used for reconstruction via the previous method.

## 1. Introduction

One of the most researched aspects in the field of computer vision relates to the human face. This is due to it being the key visual identifier that separates one person from another. Biometric recognition and analysis using 3D facial models is inherently advantageous compared to the use of 2D facial images, as it does not suffer from pose and illumination variations. However, capturing such models requires expensive and difficult-to-use 3D imaging devices which, for most tasks, are impractical or even infeasible. Moreover, many existing databases consist of only one or more 2D facial images. 3D face reconstruction attempts to bridge this gap between the 2D and 3D modalities by accurately reconstructing a 3D mesh from 2D facial images. It thus reduces the need for expensive and slow 3D imaging systems and is applicable in cases where 2D facial data are already available.

The goal of this study was to reconstruct 3D facial data from front and side-on facial images. The motivation behind using these two inputs was the fact that law enforcement agencies (LEAs) typically have datasets containing such pairs of facial images. By expanding a previous state-of-the-art single-image method and utilising synthetic data, we aimed to reconstruct 3D faces with greater accuracy. The proposed method uses a CNN to map two facial images, one front-on and one side-on, into a position map. Our network was trained on both synthetic and real data. To assess and compare our proposed method to the state-of-the-art, we utilised the MICC (Media Integration and Communication Center) Florence Dataset [1] by introducing an evaluation pipeline which aligns and calculates the facial reconstruction accuracy.

As indicated by the experimental results, the proposed method can achieve 3D reconstruction accuracy beyond the present state-of-the-art, but the fact that it requires two input images introduces extra constraints. Thus, we also present an additional approach which exploits the aforementioned procedure, while attempting to reduce the constraints that come with a two-image input network. The core idea of this latter approach is that multi-view face synthesis can be used to generate an input image pair from a single 2D image. This in turn allows the use of the two-image-input reconstruction network using only a front or side-on image.

The main contributions of this paper are: (a) The first machine-learning method for 3D facial reconstruction optimised for two facial views (front + side, as those are available to LEAs). (b) 3D facial reconstruction performance which is better than the performance of single-view methods. (c) A novel synthetic rotation methodology for facial images’ data augmentation.

The rest of the paper is organised as follows: Section 2 briefly overviews the related work in the field. Section 3 presents and analyses the proposed methodology for both cases: two facial image inputs and one facial image input. Section 4 evaluates the proposed procedures. Section 5 discusses some important issues raised throughout this work. Section 6 concludes the presented work. Section 7 records some important future challenges.

## 2. Related Work

There have been multiple publications in the 3D facial reconstruction field recently. In 2020 alone, several papers were released on the subject [2,3], especially targeting facial texture reconstruction. Several methods focused on single-view 3D face reconstruction [4,5,6,7], but there were some papers with multi-view approaches as well [8,9]. To the best of the authors’ knowledge, there have been no recent publications which specialised on using two viewpoints, front and side, in multi-viewpoint methods to deal with viewpoints from arbitrary angles.

Several 3D face reconstruction methods depend on a reference 3d morphable face model (3DMM) [10] to predict a face through regressing 3DMM parameters instead of vertex coordinates [6,11,12,13,14,15]. The volumetric regression network (VRN) [16] introduced a straightforward way to map input image pixels to a full 3D facial structure unrestricted from any face model space. The paper defined a complex network structure which predicts voxel data. The position map regression network (PRN) [4] built on the idea of mapping input data unrestricted from model space, and predicts position maps from input images. The proposed method builds upon this approach.

Alternative approaches include the exploitation of modern variations of generative adversarial networks (GANs) [5,17]; the implementation of the so-called 3D Dense Face Alignment (3DDFA) [18,19]; the utilisation of facial distinctive features [7]; and strategies such as the work in [9], where instead of directly predicting representations of 3D face vertices, a complex network is implemented which learns on the image-level losses such as skin estimation loss and the perception-level loss for deeper features of the face. The latter paper also proposed a multi-image confidence score system.

## 3. Methodology

Section 3.1 presents the proposed pipeline for reconstructing 3D facial meshes from front and side facial images. The main component of the proposed pipeline is the CNN, which is fed a concatenated image matrix and predicts the corresponding position map. Both real and synthetic training data were used to train the network. Section 3.2 presents a method for handling 3D facial reconstruction from a single facial image by computing a second facial image from a different viewpoint and feeding the image pair to the former method.

### 3.1. 3D Face Reconstruction Using Front and Side Facial Images

#### 3.1.1. Proposed Pipeline

**Figure 1:**
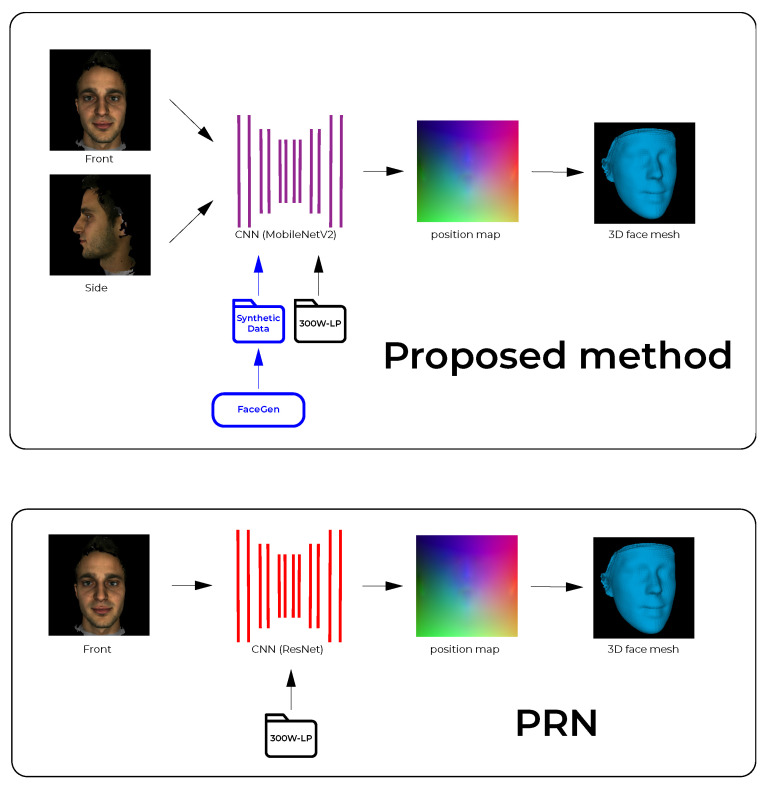
Outline of the proposed 2-input method for 3D face reconstruction (**top**), compared to the position map regression network (PRN) (**bottom**). The convolutional neural networks (CNN) in the proposed method was trained on both synthetic and real data.

The proposed pipeline builds upon the PRN implementation [4] and is outlined and contrasted to PRN in Figure 1. The PRN implementation uses a single input image and passes it through a CNN to produce a position map. The CNN in PRN was trained using only data from the 300W-LP dataset. The proposed pipeline is built around a CNN which is fed front and side images (2-input network) which produces a position map. The 3D vertices are extracted and reconstructed into a facial mesh using the face3d (https://github.com/YadiraF/face3d (accessed on 28 August 2021)) library. The proposed CNN was initially trained on synthetically generated data, exploiting the fact that 3D facial modelling software exists that can produce such synthetic data; transfer learning was then applied using real facial data from the 300W-LP dataset. Additionally, to further improve upon the PRN method, the proposed CNN uses MobileNetV2 [20] components instead of ResNet [21] components as the backbone of the network.

#### 3.1.2. Real Training Data

The training data for our network were facial image pairs, one facing the front, and one facing the left or right. The network predicts a position map which maps the face mesh vertices to one of the input images—in our case the front-facing image. The training data pairs for our network contained two images of a face as input, and one position map as ground truth.

To generate real training dataset pairs, we used the 300W-LP dataset and synthetic data from FaceGen (https://facegen.com/(accessed on 28 August 2021)). The 300W-LP dataset contains images with Basel Face Model (BFM) [22] parameters defining the shape, expression and pose. From these parameters the 3D face mesh and UV position map could be generated using the face3d library.

**300W-LP Dataset**

**Figure 2:**
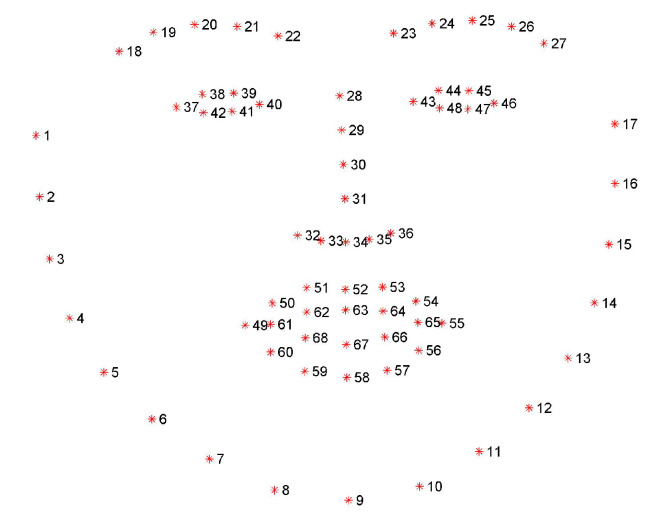
Key-point annotation in 300W-LP.

**Overview:** The 300 Faces in-the-wild challenge dataset (300W) [23] was created for a facial landmark localisation challenge in 2013. The 300W dataset includes the datasets AFW, LFPW, HELEN, IBUG and XM2VTS [23] with standardised key-point annotations, as shown in Figure 2. There were more than 3500 individuals photographed in the 300W dataset.

**Figure 3:**
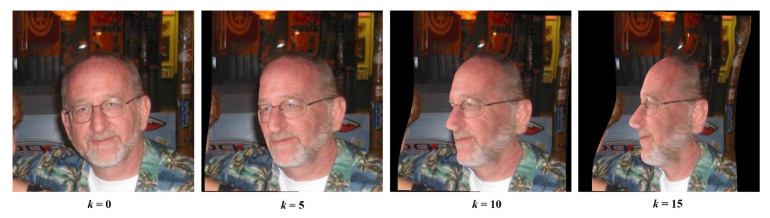
An example from the 300W-LP dataset; yaw angle rotated k number of times.

One of the limitations, other than its size, is the lack of extreme-yaw-angle poses in the dataset. The dataset lacks faces with yaw angles in the [45°, 90°] range. The 300W-LP (LP = large pose) dataset [18] is an extension of the 300W dataset which addresses this limitation. The authors fitted the faces in 300W with BFM parameters and rotated the fitted faces with yaw angles up to 90° in *k* steps, with *k* typically being in the [10, 15] range. One rotating example can be found in Figure 3. The resulting dataset is called 300W-LP and contains images and corresponding 3DMM (3D Morphable Model) parameters as described in BFM. The 300W-LP dataset consists of 122,450 image samples and serves as a good source for training data with respect to 3D face reconstruction. One issue with 300W-LP is that the BFM parameters were fitted based on only 68 key-points. As a result, the fitted 3D face meshes are not entirely accurate.

**Figure 4:**
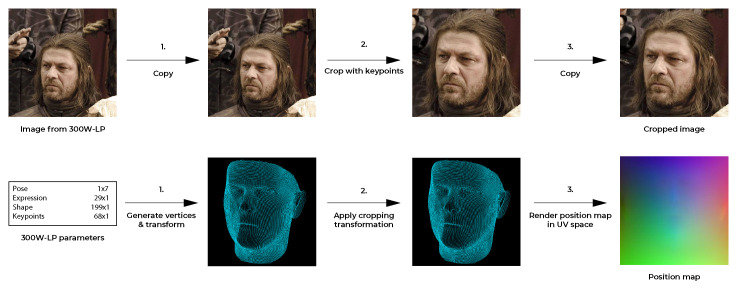
The 300W-LP training data generation pipeline. The pipeline in the top row corresponds to a pre-processing procedure and is related to the 2D data. In the pre-processing, the image is cropped based on the provided key-points and the crop parameters were kept. The pipeline in the second row represents the actual generation of the 3D training data. First, the vertices are generated and transformed to the correct pose using the face3d library. Then, the vertices are cropped according to the crop parameters that were previously extracted. Finally, the position is rendered map in UV space.

**Setup:** To generate data pairs from the 300W-LP dataset, we generated and transformed the vertices as defined in the BFM parameters, and the key-point information that each image was accompanied by. The BFM parameters were used to generate a 3D facial mesh using the mesh topology layout defined in the face3d library. Using the provided key-point information, we cropped each face and saved the cropping transformation parameters. This cropping transformation was then applied to the generated 3D facial mesh vertices to align the mesh to the new cropped image. With the transformed vertices, the position map was rendered in UV space. The pipeline was largely similar to the implementation in [4] and is visualised in Figure 4.

**Synthetic Training Data**

In addition to the real training data previously described, synthetic training data were also created and utilised. To produce synthetic training data, we used FaceGen software to render randomly generated 3D facial meshes. We then generated the position maps for the generated images by applying the rendering transformation to a corresponding 3D face mesh which was rendered into a UV position map.

The combination of the 300W-LP dataset and FaceGen resulted in more than 60,000 data sets for training our CNN.

**FaceGen Dataset**

**Overview:** Data augmentation can be useful to reduce network overfitting and to increase dataset size. An attractive way of creating a synthetic dataset is when a model of the object is available, as in the case of human faces. The SOMAnet [24] is an early example of using synthetic data to improve neural network performance in the person re-identification problem. With a human body generator, they were able to render a 100,000-instance dataset called the SOMAset. Using this data, the network was able to generalise on real-world inputs and achieve state-of-the art performance. In [25] synthetic data were used, together with real data, to train a facial recognition network. By generating synthetic faces with a face image generator, they were able to reduce the dataset bias and consequently increase the performance of their neural network. They also showed that transfer training, using first synthetic and then real-world data, increased the performance of their network. To generate synthetic faces for the 3D facial reconstruction problem, a generative model is needed, along with a way of rendering faces from different viewpoints.

**Front and Side Face Definition:** We define a front-facing image as an image of a face with a yaw angle in the [−45°, 45°] range, and a side face image as an image of a face with a yaw angle in the range [−100°, −45°] or [45°, 100°]; greater yaw angles result in occlusions of large portions of the face.

**Figure 5:**
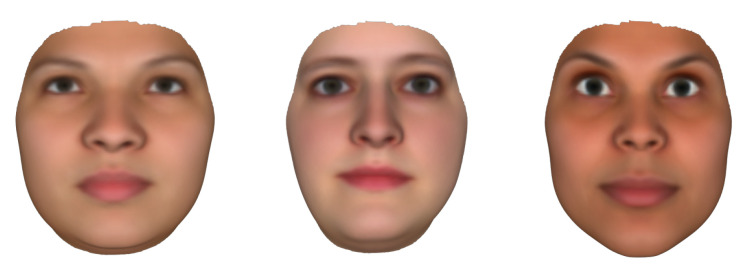
Synthetically generated FaceGen faces rendered with the Preview composite statistical appearance models (SAM).

**FaceGen Tool**: FaceGen (https://facegen.com (accessed on 28 August 2021)) is a 3D face generating software available through a license. FaceGen has created its own 3DMM using 273 high-resolution 3D face scans. The face model is parameterised through 80 dimensions of shape and 50 dimensions of colour. The FaceGen 3DMM is able to produce different mesh topologies through composite statistical appearance models (CSAMs), or just SAMs. A SAM consists of a statistical shape model (SSM) and a statistical colour model (SCM), and is able to generate random faces and render them with a mesh topology. FaceGen also provides mesh integrating tools for generating SAMs for any mesh topology layout. Example faces generated with FaceGen are shown in Figure 5.

**Constructing 3D Facial Meshes from FaceGen:** The 300W-LP 3D facial meshes were generated using a mesh topology layout, as described in the face3d library. The FaceGen 3D facial meshes needed to be converted to the same mesh topology layout. The out-of-the-box statistical shape models (SSMs) from FaceGen are only able to generate meshes with approximately 5000 vertices. The mesh topology layout is also different to the topology layout in the face3d library. To create FaceGen facial meshes with the same mesh topology, the FaceGen mesh integration tools (https://facegen.com/dl/sdk/doc/manual/meshintegration.html (accessed on 28 August 2021) was utilised. Fitting the base BFM shape’s 3D facial mesh to a FaceGen SSM generated an SSM with the same fixed mesh topology as the input BFM facial mesh. Passing this SSM, along with a FaceGen face, resulted in a 3D mesh with the face3d topology layout to be generated.

**Figure 6:**
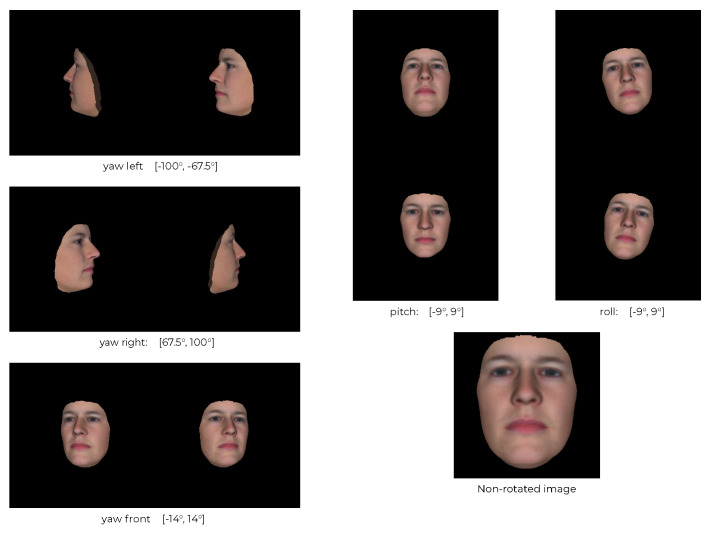
A FaceGen face with the different yaw, pitch, and roll angle ranges.

**Figure 7:**
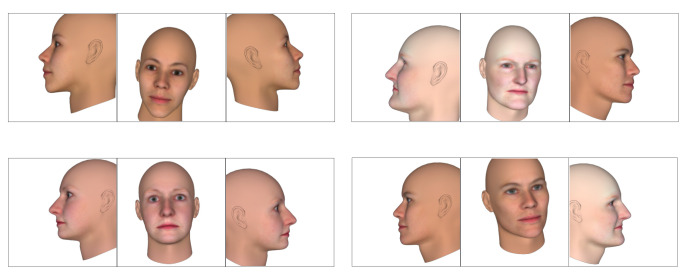
Example renderings of FaceGen faces rendered with the pose angles described in Figure 6.

**Output from Synthetic Data Generation:** We used FaceGen to generate 10 K facial sets, each consisting of one 3D mesh; renderings of the face from the front, the left and the right; and the pose settings for each rendering. The faces were rendered with different yaw, pitch and roll angles for the front, left and side facial images. The angle ranges are described in Figure 6. Example outputs are showcased in Figure 7.

**Figure 8:**
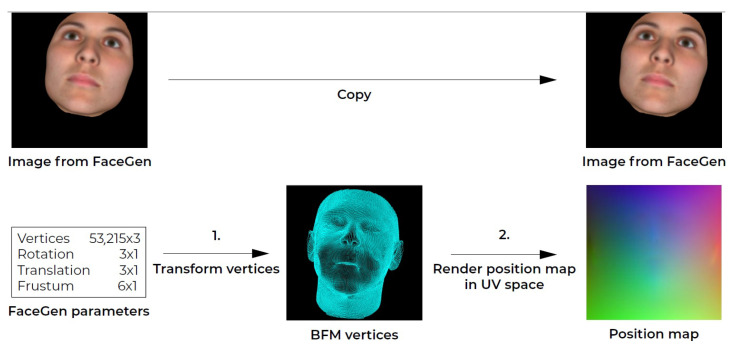
The FaceGen training data generation pipeline. A position map is generated for a facial image using the corresponding FaceGen parameters.

**Setup:** To generate training data pairs for the network, a position map for the rendered synthetic facial images needed to be constructed. This was done in two steps. First, the synthetic 3D facial mesh was transformed to the viewport coordinate system (VCS) corresponding with the facial image rendering settings. Then the vertices were rendered to UV space.The training data generation pipeline for FaceGen data is outlined in Figure 8. With the 10,000 FaceGen faces, we produced 20,000 training data pairs. Each face was rendered with front and left, and front and right facial images with the same ground truth position map, resulting in two data pairs per face.

**VCS Face Transformation:** To generate the 3D facial mesh vertex image coordinates for each synthetic facial image, the rendering settings from FaceGen were applied to the accompanying 3D face mesh. FaceGen provides the scale, translation, rotation and frustum parameters in the rendering settings. The scale, translation and rotation were applied to the 3D mesh through matrix multiplication. To take the vertices from the eye coordinate system (ECS) to the canonical screen space (CSS), an extended viewing transformation was applied. To take the vertices from CSS to the viewport coordinate system (VCS), we applied a viewport transformation [26]. A vertex point $X_{w}={\left[x_{w},y_{w},z_{w},1\right]}^{T}$ in the mesh was converted into VCS using Equation (1). The $M_{WCS\to ECS}$ transform includes scaling, rotation and translation.

(1) $$\left[\begin{array}{c} x\\ y\\ z\\ w \end{array}\right]=M_{CSS\to VCS}^{VIEWPORT}\cdot M_{ECS\to CSS}^{PERSP}\cdot M_{WCS\to ECS}\cdot X_{w}$$

**Figure 9:**
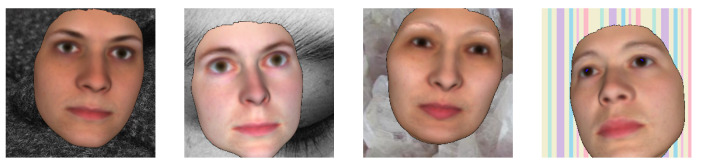
FaceGen facial images with random textures as backgrounds.

**Applying Random Background Images:** To further improve generalisation, we applied random backgrounds to the facial images generated by FaceGen. Inspired by the face generator in [25], a random texture was chosen from the Describable Texture Database [27] and added to a FaceGen image. Example faces with random texture backgrounds are shown in Figure 9.

#### 3.1.3. CNN Implementation

**Figure 10:**
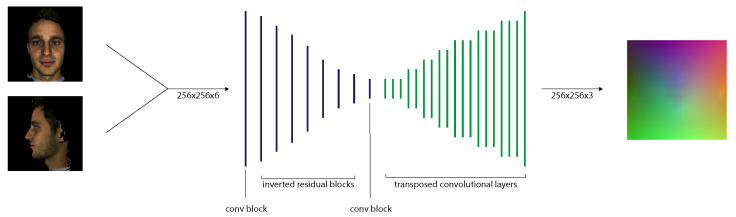
Our proposed 2-input CNN architecture for 3D face reconstruction.

To predict position maps from input images, we implemented a new CNN with Keras (https://keras.io/ (accessed on 28 August 2021)) (see Figure 10). We used the original PRN implementation as a starting point for our network architecture. We adjusted it to allow a concatenated 256×256×6 image input and replace the ResNet modules with inverted residuals. We trained the network on the synthetic data generated by FaceGen before training it with data from the 300W-LP dataset.

**Input and Output**

**Figure 11:**
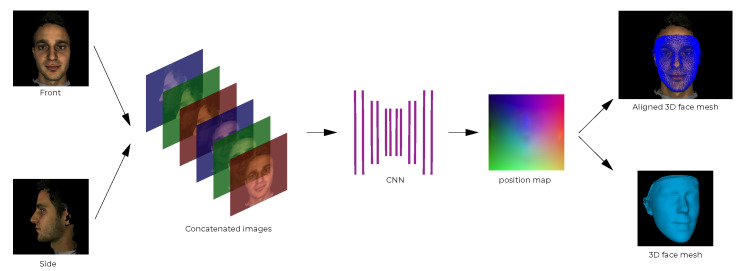
The proposed 3D facial reconstruction pipeline using an image pair.

The size of both input images is 256×256×3 for the height, width and colour channels, respectively. This is the same as the size used in PRN. As we use front and side facial images instead of a single image, we concatenate the images, expanding the original image colour channel dimensions, resulting in an image matrix of size 256×256×6, as visualised in the second step of the pipeline in Figure 11. The position map is of size 256×256×3, the same as in PRN, which means that the position map is capable of containing 256×256= 65,536 vertices, which is enough to define a 3D facial mesh accurately [4].

**Network Architecture**

We employ an encoder-decoder network structure to map the input image to the output position map. The encoder part of our network consists of 1 convolutional layer, followed by 4 inverted residual layers and finally 1 convolutional layer. The inverted residual layers are internally repeated 1–4 times. The decoder part of our network consists of 17 transposed convolutional layers. The network layers are listed in Table 1. The network has a total of 11,791,273 parameters and occupies 154 MB.

We chose the MobileNetV2 inverted residual blocks (instead of ResNet blocks used in PRN), as the MobileNetV2 architecture is more lightweight and performs better in image processing tasks [20]. The inverted residual blocks were also easy to implement, as there are several implementations available (https://github.com/d-li14/mobilenetv2.pytorch) (https://github.com/xiaochus/MobileNetV2 (accessed on 28 August 2021)). We set the filter sizes of each layer to reduce the 256×256×6 input to 8×8×512 feature maps, similarly to PRN. The kernel size for the inverted residual blocks is 3, and for the transposed convolutional layers it is 4. We use zero-padding and ReLU as the activation function for 16 layers of our decoder network, and sigmoid for the final one.

We use the Adam optimiser with the same loss function and weight mask as in the original network implementation.The batch size is set to 16, and we found the best learning rates and learning halving rates empirically for each training step.

**Training on synthetic data**

**Figure 12:**
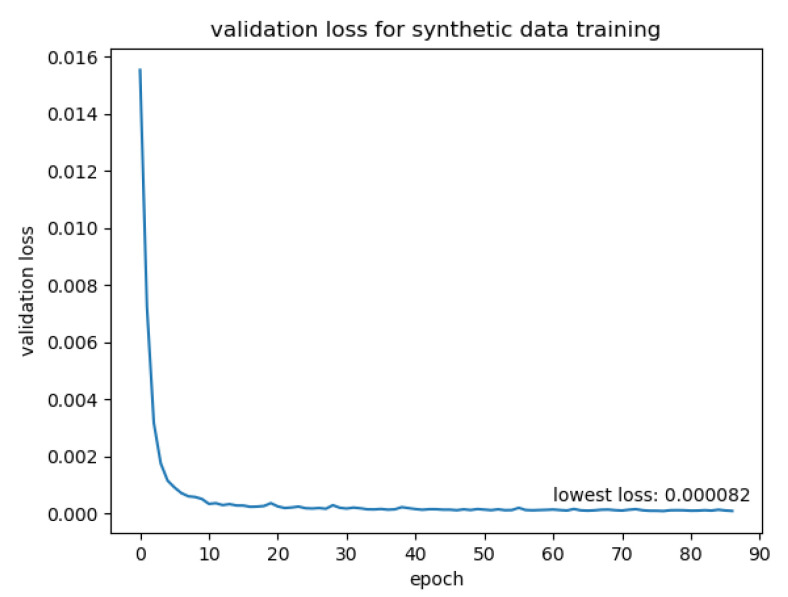
Validation loss over synthetic data training.

We trained the network on the 20,000 synthetic data pairs. We split the training data pairs into training and validation sets for training evaluation. The data pairs were shuffled before each epoch to randomise the batches. The training data were augmented before training by rotation, colour channel scaling and image dropout, as described in Section 3.2.2. The rotation was set to be in the [−45°, 45°] range; the random colour channel scale was between 0.9 and 1.2. We trained the network with an initial learning rate of 0.0001. The learning rate was halved every 5 epochs. After each epoch, we calculated the loss on the validation data. If the validation loss had not decreased for 10 epochs, the learning was suspended. We trained the network on a computer with an Intel Core i7-8700K CPU (Intel, Chongqing, China) and a NVIDIA RTX 2080Ti GPU (NVIDIA, Hsinchu, Taiwan). The validation loss is plotted in Figure 12.

**Transfer Learning with 300W-LP data**

**Figure 13:**
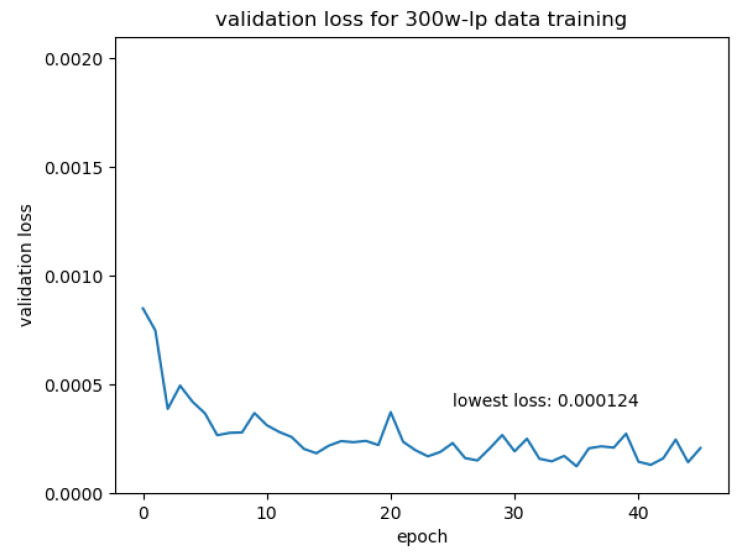
Validation loss over transfer training.

To train the network on “real” data, we restored the model weights from the model trained in the previous section. We generated training data pairs from the 300W-LP dataset using the front and side definition given earlier. For each 300W-LP face, we selected all images with a face yaw pose within [−45°, 45°]. We paired each of these front-facing images with one image of the same face with a yaw pose within the [−100°, −45°] and [45°, 100°]. These images were then augmented the same way as the synthetic data before being fed to the network. We set the initial learning rate to 0.00001 and trained the network. The learning rate was halved every 5 epochs. The validation loss is plotted in Figure 13.

### 3.2. 3D Face Reconstruction Using a Single Facial Image Input

The main idea here is that multi-view face synthesis can be used to generate a synthetically rotated facial image from a real one, and then the previously presented two-image-input reconstruction network can be used.

For the multi-view face synthesis, a slightly modified version of Rotate-and-Render [28] was used. This is a GAN-based unsupervised method for synthetically rotating the yaw angle of a 2D face in the [−90°, 90°] range. The key feature of this network is how it manages to train without any ground truth image to compare its output to. This is quite important, as virtually every dataset that incorporates large-pose images either had its images taken in heavily controlled environments [29], which affects generalisation, or has synthetic images [18], which propagates the error from dataset to network.

The training process of this network is as follows: Reconstruct the image into a 3D model, rotate it and render it in 2D. When this first phase of training has concluded, the whole process is reverted by reconstructing the image to 3D, rotating it back and rendering it in 2D. By doing this, the model can compare the back-and-forth rotated face to the original image as a form of self-supervision.

#### 3.2.1. Proposed Pipeline

**Figure 14:**
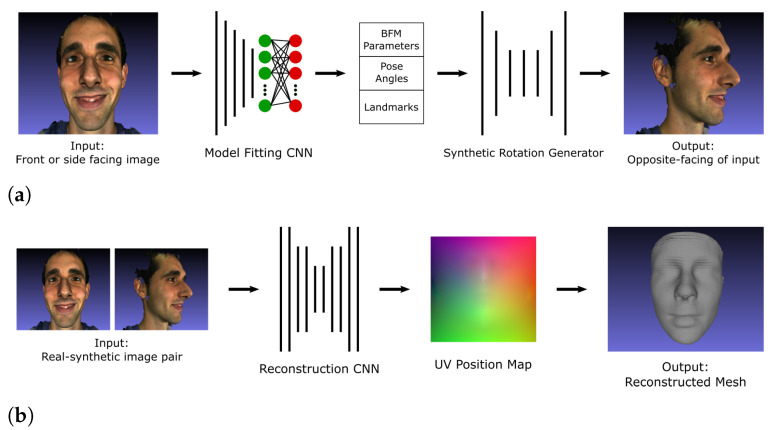
The proposed pipeline for synthetic facial image rotation: (**a**) Synthetic rotation pipeline, from one real facial image input to a synthetically rotated version, (**b**) Reconstruction pipeline from 2-inputs, one real and one synthetically rotated (as in (**a**)).

There are two main components that make up the proposed pipeline for 1-input 3D facial reconstruction: a network that rotates a given facial input image, and a network that reconstructs a 3D face from the original and synthetically rotated image input pair. For the latter part, the method described in Section 3.1 is used. The proposed pipeline is illustrated in Figure 14.

**Synthetic Rotation**

The idea for this module was inspired by the large-pose synthesis method [18] applied to images in the 300W-LP dataset. However, due to its slow inference time, Rotate-and-Render [28] was chosen instead for its good performance and reasonable run time.

Rotate-and-Render was re-addressed so as to be inter-operable with the reconstruction system. The way Rotate-and-Render originally worked was by first regressing BFM parameters for a batch of images. Those BFM parameters were then turned into a 3D facial model and rotated in 3D space. Finally, this rotated model was rendered back into 2D. These processes were performed separately, which means that the user would have had to manually call multiple processes to get a rotated image, and finally call the reconstruction network to get a 3D face. To amend this, the rotation network was modified to do everything in one pass.

**Figure 15:**
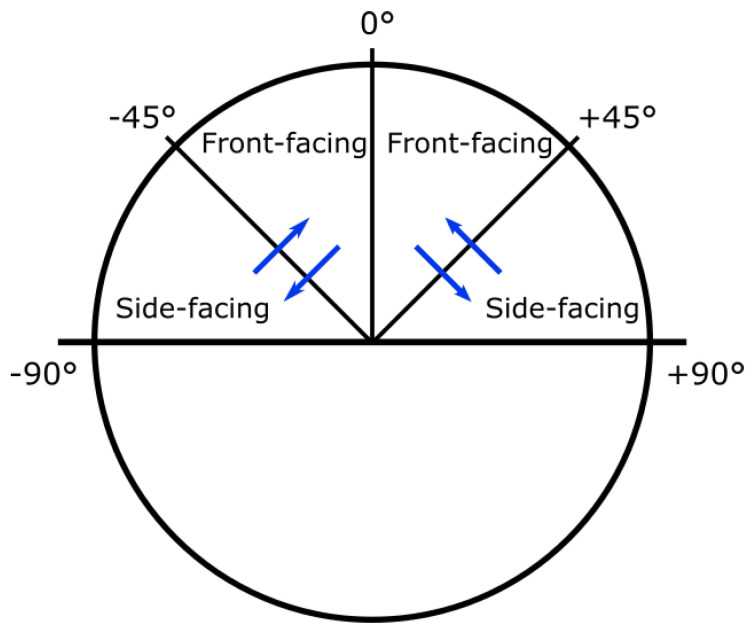
A bird’s-eye view of the human face. The blue arrows indicate the directions the automatic rotation function will rotate the face in, given the right yaw angles.

In addition, a procedure that automatically determines which direction to rotate the image has been created. Initially, in Rotate-and-Render, the user had to manually input the desired yaw-angle of the rotated image. For the purposes of this system, a function that detects whether the input image is front- or side-facing and rotates it accordingly is utilised. The rotational directions for different yaw-angles are depicted in Figure 15. The angle by which the face is rotated can be adjusted, but for this particular experiment it was fixed to 30°. As long as the pose of the input is in the [−90°, 90°] yaw range, this procedure will yield a valid rotation.

#### 3.2.2. Training Data

The dataset used for training was synthetic and generated by FaceGen, as previously described. Each FaceGen model—or subject—in the dataset was rendered at yaw angles [−90° ±67.5°, ±45°, ±22.5°, 0°], plus a random noise factor sampled uniformly from the range [0°, 22.5°]. Each rendering also has a random roll and pitch angle in the range [−25°, 25°].

**Figure 16:**
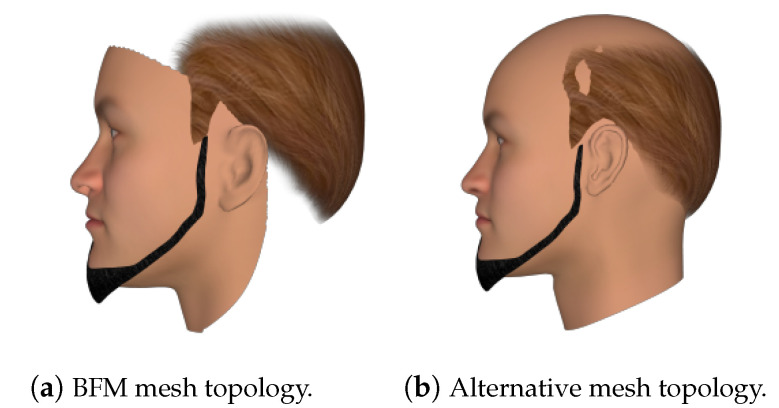
Rendering a FaceGen subject from a large yaw anglewith two different mesh topologies.

When rendering this way, there are a few obvious flaws. First of all, while BFM meshes are fairly detailed, it also only models the identifiable parts of the human face—that is, the front. This might not be a problem for front-facing images, but for large yaws, this leads to rendered images looking fairly strange, as part of the back and side of the head is not modelled. Secondly, since the top of the head is missing from these models, it can cause a situation where the hair looks like it is floating, depending on the hairstyle. Both of these cases are illustrated in Figure 16a.

To fix these issues, an alternative rendering method was used. Instead of using a BFM mesh for rendering, another mesh topology that models the whole face was employed. An example can be found in Figure 16b. The BFM model still needed to be generated though, as it was used to generate the ground truth UV position map. As for that, the new base mesh was aligned with the BFM base mesh, so that the positions of the vertices on the rendered image matched up with those on the position map.

Initially the idea was to also include the 300W-LP dataset in the training process, by including synthetically rotated versions of the facial images. However, the synthetic rotation network had issues recognising some of the larger-pose faces in 300W-LP due to their synthetic nature, and we opted out of using this dataset.

**Pre-processing**

A pre-processing step that was beneficial in both the training and testing phases was cropping. When running the network on images in-the-wild, the sizes and positions of the face in the image might vary wildly. Cropping ensures that the network can always expect a centred face of a certain scale as input. In contrast, if cropping is not performed, the network would have to account for variations in the position and scale of the face, in addition to actually performing the reconstruction. This is quite undesirable, as it would add unnecessary parameters to an already difficult problem. Another benefit of cropping is that it discards most non-facial data. It is essential that the input contains as much relevant information as possible, especially at such limited resolution.

To perform the cropping, a lightweight face recognition network included in the dlib (http://dlib.net/ (accessed on 28 August 2021)) library was used. One might argue that if the reconstruction network increases in complexity, it might be able to do the work of the recognition network and perform reconstruction in one pass, but there are good reasons for not doing this. First, creating datasets for two networks with well-defined tasks is far easier than that of a single complex structure. Second, training with a single loss-function for what is essentially two different tasks might make it unclear what is working and what is not.

Only the non-rotated image is cropped. This is due to the face detector having a difficult time recognising faces on synthetically rotated images. It is also worth mentioning that the output from the synthetic rotation network is fairly constant in the sense that it is always centred and more or less on the same scale. There is no real performance loss from not cropping the synthetically rotated image.

**Data Augmentation**

**Figure 17:**
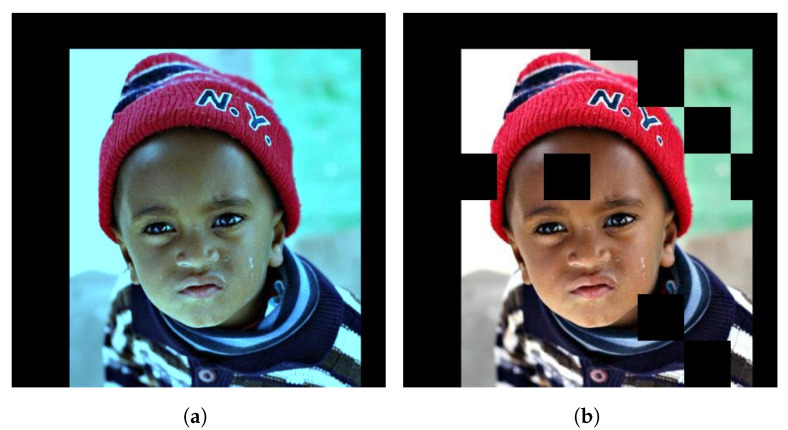
A subject from 300W-LP with different augmentations: (**a**) Colour channel scaling, (**b**) Dropout.

Two data augmentation techniques were applied, the first of which was colour channel scaling. In computer graphics, the most widely used method of representing colour is the RGB model. Colour channel scaling means that each of these channels is multiplied by a different scalar. For this implementation, the scalar is random and uniformly sampled from [0.6, 1.4]. An illustration of what this might look like can be found in Figure 17a. In practice, what colour channel scaling achieves is modifying the overall colour of the image without altering the shapes. Ideally a 3D reconstruction network is able to regress a model based on the contour and shapes of a human face. This is exactly why colour channel scaling is such a good fit in this particular instance. Due to its shape-preserving nature, it makes sure that no discrepancies between input and ground truth are present. It also penalises reliance on colours, increasing the generalisation between different lighting conditions and camera settings.

The other augmentation that was applied is called dropout, and might not be as intuitive as the former. What dropout essentially does is ignores certain neurons during the training process. The neurons that get ignored change with each iteration of training. Dropout is often applied inside a neural network at different layers during a forward pass, although in this case it is only applied on the input layer. Figure 17b illustrates the application of dropout on an image. Dropout leans heavily into the definition of regularisation, which defines it as reducing the coefficients of certain input parameters of the model. Intuitively, this means that the model will learn not to be too fixated on specific pixels, but rather on the image as a whole.

#### 3.2.3. Training Procedure

The network entered the training stage with initial weights from the 2-input image network. Then the image was cropped and synthetically rotated. Then the ground truth UV-position map was calculated from the ground truth BFM FaceGen mesh. The resulting input image pair and ground truth UV map were saved for training. Colour channel scaling and dropout was applied to the input image pair right before loss, and gradients were calculated during training. Mean squared error was used as the loss function.

One issue was whether to synthetically rotate after every augmentation had been applied or not. The former would definitely be more realistic, but there is a big downside to this approach, which is that the rotation network is fairly slow, and since augmentations are applied on a per-epoch basis it would mean that the rotation network would have to be run at a rate proportional to the number of epochs. Thus the decision was made to only run the rotation network once for every image and save the results.

## 4. Evaluation

We assessed our networks using the MICC Florence dataset. This Section presents the evaluation dataset, the evaluation pipeline and the performance results for our networks. The performance of our networks is also compared to the performance of the network in [4] (PRN).

### 4.1. Evaluation Data: MICC Florence Dataset

We used the MICC Florence dataset [1] as the evaluation dataset. The dataset consists of 2D images, videos and high-resolution 3D scans of 53 subjects. The images, videos and 3D meshes for each face are stored in separate folders. Each subject was scanned from multiple angles. For our evaluation, the frontal scans were used. More specifically, the .obj file and the corresponding texture data from the Model/frontal1/obj folder for each subject were used. Some of the subjects had facial hair, which was included in the 3D scans. The 3D faces used to train the proposed network and PRN did not have facial hair, which increased the error for subjects with facial hair. As the facial hair error situation is similar for both PRN and the proposed method, these subjects were not excluded.

### 4.2. Evaluation Metric

The goal of the evaluation metric was to evaluate the network’s ability to reconstruct 3D facial meshes; thus, the evaluation metric measured the difference between two facial meshes. We employed the normalised mean error (NME) of the euclidean distance between the points of the predicted and ground truth 3D mesh to be the evaluation metric. The error function is defined in Equation (2). ${\left||\left(p_{i}-q_{i}\right)|\right|}_{2}$ denotes the euclidean distance between two points, and *d* denotes the normalisation factor. Points $p_{i}$ and $q_{i}$ are are the corresponding points on the ground truth facial mesh and the predicted facial mesh, as indicated by the ICP algorithm [30]. The normalisation factor was set to be the bounding box size of the predicted 3D facial mesh.

(2) $$NME=\frac{1}{N}\sum_{i=1}^{N}\frac{\left|\right|\left(p_{i}-q_{i}\right){\left|\right|}_{2}}{d}$$

### 4.3. Evaluation of the Two-Input Pipeline (Front and Side)

#### 4.3.1. Evaluation Procedure

We rendered front and side facing images from the MICC Florence dataset, predicted the corresponding position maps and evaluated the output facial meshes. The images were rendered from extracted .obj files in MeshLab (http://www.meshlab.net/ (accessed on 28 August 2021)) with orthographic projection. After generating a 3D facial mesh from the predicted position map, we fit the predicted 3D facial mesh to the ground truth 3D mesh provided in the MICC Florence dataset. We aligned the meshes using an implementation of the ICP algorithm (https://github.com/ClayFlannigan/icp (accessed on 28 August 2021)). If necessary, an initial alignment was passed to the algorithm. The ICP implementation output the rotation matrix and translation vertex as a homogeneous transformation matrix, which maps a point set *X* to a point set *P*. After applying this transformation matrix to the predicted 3D face mesh, we calculated the evaluation metric.

#### 4.3.2. The Performance of the Synthetically Trained Network

**Figure 18:**
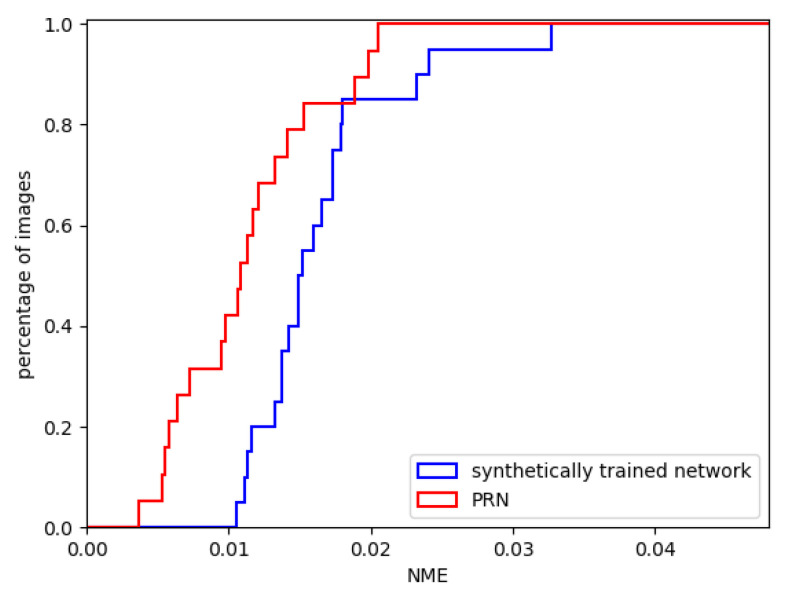
Cumulative error distribution (CEDs) for the synthetically trained 2-input network and PRN.

**Figure 19:**
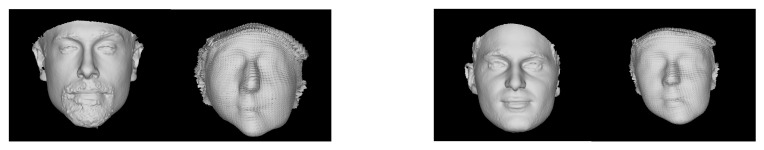
Two 3D facial meshes from the MICC Florence dataset (**left** part of each pair) compared to the reconstructed 3D facial meshes made by the synthetically trained 2-input network (**right** part of each pair).

First, the performance of our synthetically trained 2-input network was evaluated. We ran 20 randomly faces from the MICC Florence dataset through the evaluation pipeline using our synthetically trained network. We visualised the results by utilising a cumulative error distribution (CED) curve in Figure 18. The same figure illustrates a direct comparison of our proposal against the PRN, which is the most popular reconstruction technique of the state-of-the-art. The mean NME is presented in Table 2 and is compared against the current state-of-the-art. Figure 19 shows two exemplary predicted 3D facial meshes together with the ground truth meshes.

**Figure 20:**
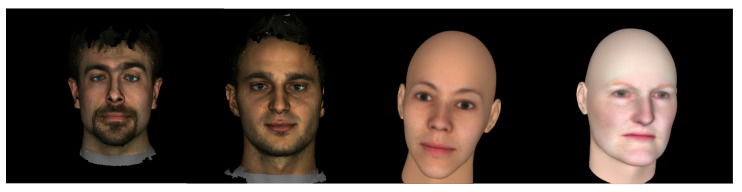
Random images from MICC Florence and FaceGen datasets. The two images on the left are from MICC Florence and the two images on the right are from FaceGen.

It appears that the synthetically trained network performed slightly worse than PRN on the MICC Florence dataset. By looking at the two exemplary outputs of the networks in Figure 20, the shortcomings of the synthetically trained network become apparent. The reconstructed mesh has an asymmetrical face shape compared to the ground truth facial mesh, and there are few visual similarities. There are also artefacts along the top seam edge of the mesh.

A likely explanation for the network’s inability to reconstruct convincing 3D meshes is the fundamental difference between the network’s training data and the MICC Florence test data. Example images from the test dataset and FaceGen are shown in Figure 20. The lack of realistic hair, expressions, accessories and skin texture likely reduced the generalisation of the synthetically trained network. The facial asymmetry observed in Figure 19 could be a result of overfitting. If the synthetic training data consistently contains faces with a narrow jaw shape, the network could be unable to generate output meshes with wide jaws. Lastly, the artefacts in the reconstructed facial mesh are likely the results of a mechanism connected to the generated weight mask used to calculate the loss function. The artefacts occurred at the outermost vertices covered by the weight mask. The same artefacts were also found to some degree in the reconstructed meshes from PRN.

#### 4.3.3. The Performance of the Transfer Trained Network

**Figure 21:**
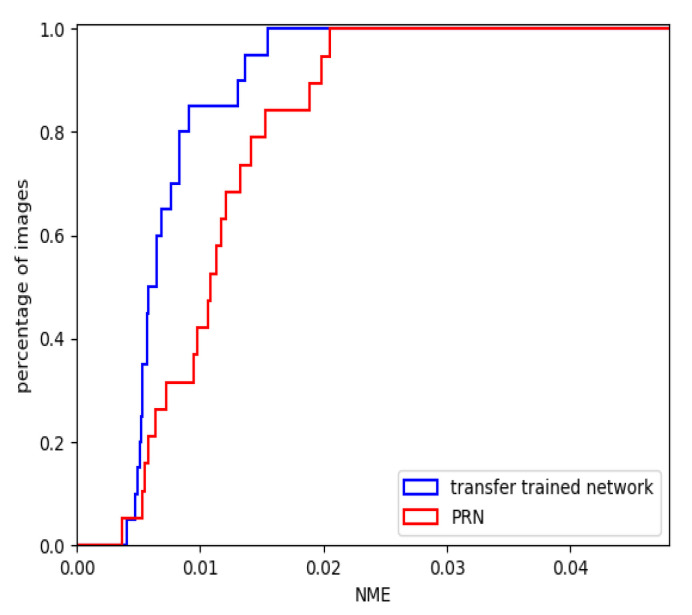
CED for transfer trained 2-input network and PRN.

**Figure 22:**
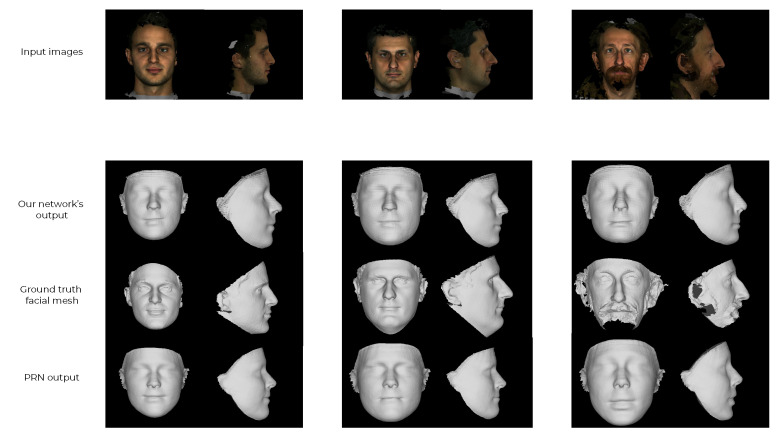
Examples of meshes that were reconstructed using three faces from the MICC Florence dataset. The top row shows the input images for the networks. PRN uses a single front-facing image as input, whereas our network is fed both the front and side images. The transfer trained network output is shown in the second row, the ground truth facial mesh in the third row and the PRN output in the fourth row.

To evaluate the transfer trained network, we again ran the evaluation pipeline using the same 20 faces that were used in the previous section. The CED curves for our transfer trained network and PRN, being the most popular state-of-the-art network, are plotted in Figure 21. The mean NME is presented and compared against more state-of-the-art techniques in Table 3. Figure 22 shows the constructed 3D facial meshes from three example faces using both the transfer trained network and PRN.

The CED curve in Figure 21 and mean NME in Table 3 show that our transfer trained network was able to reconstruct more accurate 3D facial meshes than PRN. More than 80% of the reconstructed faces using the transfer trained network had NME lower than 0.01, whereas approximately 40% of the faces reconstructed using PRN had NME lower than 0.01.

When examining the exemplary reconstructed 3D facial meshes in Figure 22, the difference is not as clear. By comparing the transfer trained network’s output with the ground truth facial mesh, we only see some similarities. The most distinct facial features were not accurately reconstructed. The reconstructed meshes using our network are more similar to the meshes reconstructed using PRN. There are some differences between our network and PRN around the nose and jawline. The PRN reconstructed similar noses for all the example faces, whereas our network was able to reconstruct slightly more varied nose shapes. The difference is clearest in the rightmost face in Figure 22. The artefacts found in the synthetically trained network largely disappeared. Both networks also predicted faces without facial hair. The lack of facial hair was a result of the training data, as the synthetic and 300W-LP datasets provide 3D faces without facial hair as ground truth.

The reconstructed faces are visually similar to each other, but are ultimately unable to accurately reconstruct distinct facial structures. One probable explanation for the similarities between the reconstructed 3D facial meshes of our network and PRN is the training data foundation. The transfer trained network was trained on data from the 300W-LP dataset. The ground truth faces in 300W-LP were generated from 68 2D key-points using a CNN. By training a network on 300W-LP, we risked simply predicting the output of the CNN used to generate the 300W-LP faces. This might also explain the inability of our network to further utilise the additional side image input to greatly improve the performance of our network.

### 4.4. Evaluation of The One-Input Pipeline

**Figure 23:**
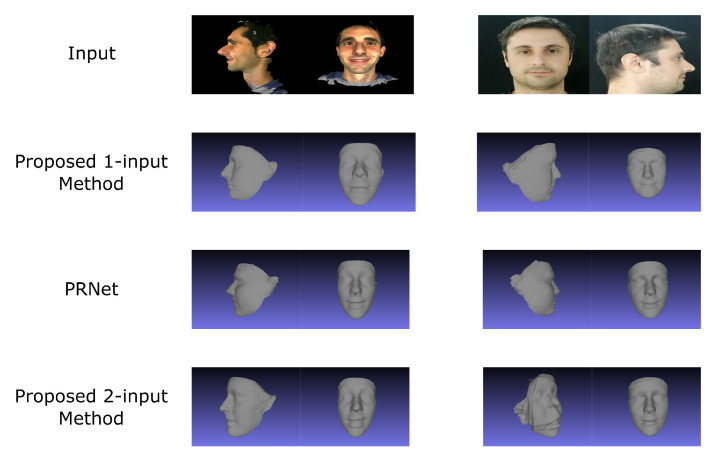
Reconstruction results from different models. The image to the left is from MICC Florence, and the one on the right is from a proprietary NTNU dataset.

**Figure 24:**
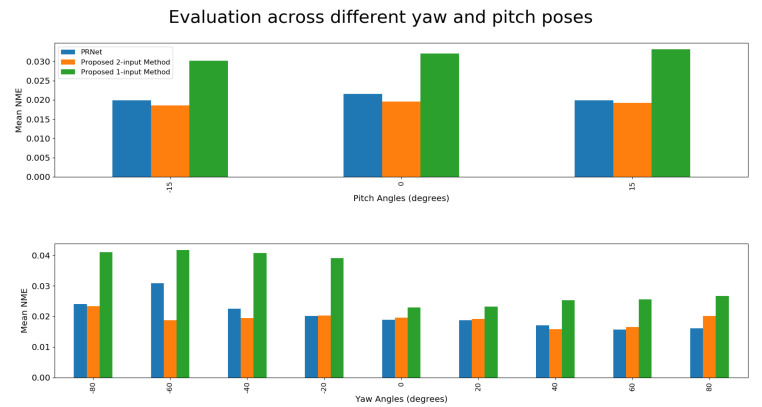
Mean NME of different models at different angles.

The evaluation was performed by reconstructing a face and then aligning it with the ground truth model using ICP (Iterative Closest Point). This time, an additional proprietary database, owned by NTNU, was recruited for conducting experiments. The joint results are illustrated in Figure 23. NME values can be found in Figure 24 and Table 4.

Surprisingly, the best performing model was not the one that was trained specifically with synthetic rotation in mind, but rather the one trained for the purpose of using a real front and side facing image. There were a few issues with the synthetic dataset that led to such poor performance. First of all, it seems as if the synthetic data is simply not realistic enough to provide top-of-the-line results. This was a real concern when looking at the performance of the synthetically trained network described in Section 4.3.2.

**Figure 25:**
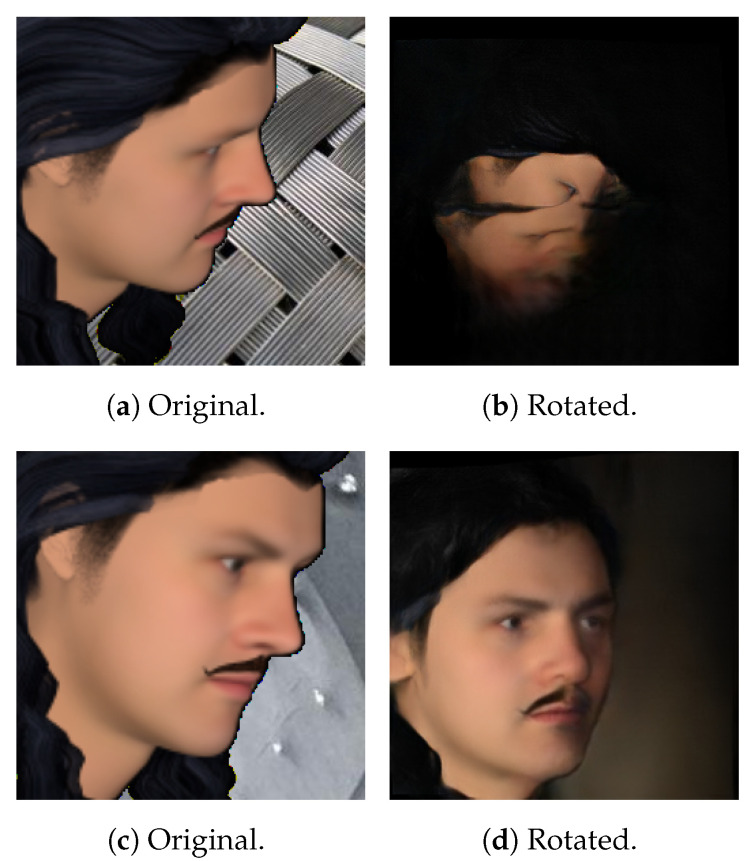
The same subject from two different angles and the corresponding attempts at synthetic rotation. On the top is an unnatural result and on the bottom a “proper” one.

The poor level of detail on FaceGen subjects propagated to other parts of the system as well. The synthetic rotation network seemingly had very inconsistent results, depending on the subject composition and pose. This is illustrated in Figure 25. Synthetic rotation has a tendency to generate results such as that in Figure 25b when the pose gets large, but this was not nearly as frequent with real data as it was with the FaceGen dataset. This creates a discrepancy between the data it is trained on and the data it is tested on.

Lastly, the model was not able to reconstruct facial hair properly. It seems that the model was not able to learn to model it properly, probably due to restrictions of the synthetically generated facial-hair data.

The fact that the proposed 2-input network was able to perform better than PRN, even if only slightly, demonstrates the capability of the system if trained on “real” datasets such as 300W-LP, especially since it is prone to some high-error outliers, as is illustrated in Figure 23, bottom row, second column.

Outliers such as these are thought to stem from a combination of flaws within the system, the first being sub-optimal performance of the synthetic rotation network at large angles. Secondly, there was a discrepancy between the input expected by the proposed 2-input network and the actual inputs that were received. As explained in Section 3.1.2, the training process for the two-input-image network is such that it expects a side-facing image concatenated to a front-facing one, while always regressing the UV position map for the front-facing image. On the other hand, the proposed single-input-image network generates the input pair in such a way that the synthetically rotated image is concatenated onto the real one, meaning that the image in colour channels 1–3 could be either front- or side-facing. Similarly, the regressed position map could either be that of a front-facing image or that of a side-facing image. While the former issue might end up being quite difficult to solve, the latter would in theory only be a matter of adjusting the order of images in the input pair during training.

Due to the impressive performance of using 300W-LP with weights that were meant for a different network, it is believed that going forward this will be the preferred training dataset for this system. To circumvent the issues detailed in Section 3.2.2, it will likely be better if synthetic rotation is not performed during training, and instead, one could use front and side-facing images from 300W-LP as an input pair. The image that is supposed to be the synthetically rotated image in the image pair would need to be modified to better mimic the output of the synthetic rotation network. A few examples could include: using the same background colouring method as Rotate-and-Render, setting pitch pose to zero and cropping/scaling the image in the same way Rotate-and-Render would. The synthetic rotation network could also be modified to better mimic the data from 300W-LP. The most notable changes would be to retain the pitch pose when rotating instead of setting it to zero, and cropping the output image in the same way as data from 300W-LP would be cropped.

### 4.5. Potential Sources of Error

#### 4.5.1. MICC Florence Dataset

**Figure 26:**
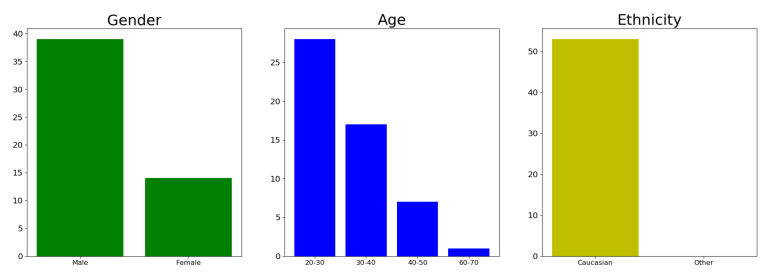
Demographic of subjects captured in the MICC Florence dataset.

During evaluation, it is important that the dataset is as detailed and realistic as possible, but also that it represents the general populace in a proper manner. The concern with MICC Florence is that certain demographics are over-represented, and some are non-existent. Histograms illustrating the demographics of the evaluation dataset are found in Figure 26.

The issue with data bias in an evaluation set is that it will not be able to capture the proper level of generalisation for the models that it evaluates. A model that only performs well on Caucasians would seemingly get great results when evaluated on this particular dataset, even though it would have bad generalisation across races. This in turn might create an inaccurate picture of the performance of a model.

Another issue with this dataset is that there is no set standard for rendering the models. This means that there might be deviations between the evaluations of different experiments. As an example, the NME of PRN in this particular paper is quite a bit lower than what was found in [4]. The differences in rendering that may cause these discrepancies might be related to lighting, position of the face in the image, scale, etc. Additionally, since the models were not made to look straight forward, the rendering of meshes at certain poses required some approximations, which introduced another potential error. Since every model was evaluated with the same number of renderings per experiment, this is not necessarily a problem. However, it could cause some confusion when making comparisons with other works.

#### 4.5.2. ICP Alignment

The evaluation pipeline utilises ICP to align a reconstructed 3D facial mesh to a ground truth mesh for evaluation. ICP requires an initial alignment to align two point sets correctly. By saving the initial alignment parameters for each face reconstructed using PRN to the correct ground truth 3D facial mesh, this source for error is largely minimised. As PRN and our network produced 3D facial meshes with vertices in the same value range, the initial alignment was valid for both meshes. However, if a reconstructed facial mesh is not similar enough to the ground truth mesh, the face will not be correctly fitted. This fitting error is difficult to avoid, but with the initial alignment, most faces were fitted correctly. Most of the faces that were reconstructed using the synthetically trained network and some of the faces with the highest NME in Figure 21 probably suffered from bad alignment. As the initial alignment parameters were found using PRN, any ICP fitting disadvantage should have been similar for PRN and our networks.

### 4.6. Qualitative Evaluation of Synthetic Rotation

**Figure 27:**
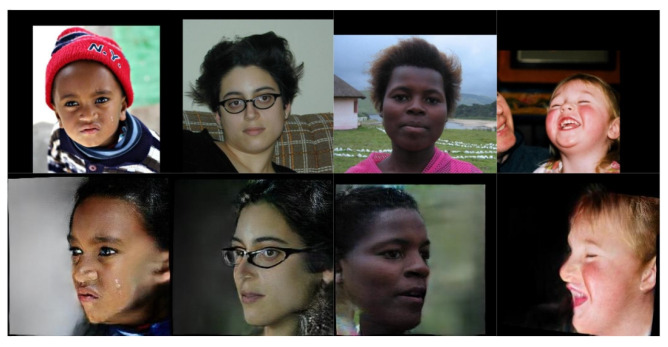
Synthetic rotations of images in 300W-LP.

The modified Rotate-and-Render seems to be better than the original version. The network performed best when the input image was a slightly rotated front-facing image, as this is the angle that least occludes the parts of the face that are included in the synthetic rotation. The network also seemed to gain in performance the closer that pitch rotation was to zero. This was most likely due to the fact that the network sets pitch rotation to zero degrees for its output, and as a result, the more rotation the original image has in this direction, the more the network has to rotate. A few example images are shown in Figure 27.

## 5. Discussion

As described in Section 4, the performance of our initial, synthetically trained network was worse than that of the state-of-art, PRN. This was partly to be expected, since the evaluation dataset consisted of real data. However, by using the synthetically trained network weights as initial weights and performing transfer training on real data, the proposed network outperformed PRN (using two input facial images). Increasing the realism of our synthetic dataset is expected to boost its performance both on synthetic data and on real data (after transfer learning). Some obvious ways to this end are to apply realistic hair, facial expressions and skin textures.

In the 3D facial reconstruction field, the lack of accurately labelled training data increases the importance of synthetic data. Labelling 2D images with corresponding 3D facial features is time consuming and difficult. 300W-LP uses a CNN to create 3D faces using as input 2D facial images and 68 key-points. While this simple approach of generating labelled training data produces many data pairs quickly, it is not entirely accurate. With synthetically generated data the ground truth 3D facial mesh is always accurate.

To generate synthetic training data, we utilised FaceGen. FaceGen provides powerful tools for generating 3D faces, but the output from a simple FaceGen statistical appearance model without expression, hair and detailed skin texture is not good enough to train a generalised network. Improving the photo-realism of the proposed synthetic data generation pipeline output could be useful. Additionally, increasing the amount of data, both real and synthetic, should also result in increased performance.

An interesting question is whether the current state of the reconstructions is sufficient for 3D face recognition performed by LEAs. In a separate experiment, conducted on a small but difficult proprietary dataset that consisted of on-the-fly 3D captures, we observed a 15% decrease in performance when using reconstructed 3D faces in the probe set (from front and side facial images) compared to real 3D scans. We are thus led to believe that there is significant scope for further improvement in the quality of 3D facial reconstructions from 2D facial images. At the current state of performance, it could be used as a component in a biometric fusion scheme but is unlikely to be useful as a stand-alone modality.

The run-time of the rotated image synthesis network is on average 0.6 s per image on an RTX 2080TI. This makes it unsuitable for applications such as video, where a stream of images needs to be reconstructed in real time. Even with the release of a new generation of consumer-grade GPUs (Graphics Processing Unit), it seems unlikely that this system will achieve real-time performance.

## 6. Conclusions

In this paper we have proposed and evaluated a new method which reconstructs a 3D facial mesh from front and side images using a CNN. The CNN was built on the network proposed in [4] and achieved a lower mean NME on the MICC Florence dataset. Using a concatenated image matrix as input, the network predicts a position map, from which a 3D facial mesh is generated. Finally, the network was initially trained on synthetic data generated using our proposed synthetic data generation pipeline, and then transfer learning was applied using real data from the 300W-LP dataset.

A scheme which exploits the aforementioned network in order to perform reconstruction in cases where only one facial image is available, was also proposed. To this end, it built upon the multi-view synthesis approach proposed in [28], so as to create synthetically rotated facial images which act as the second components of the pairs of images to be fed to the two-image network proposed above.

## 7. Future Work

One main issue addressed in this paper was the lack of accurately labelled training data for the 3D facial reconstruction problem. An improvement would be to utilise accurate real training datasets. Another improvement would be to further increase the realism of the proposed synthetic data generation pipeline, through adding hair, facial expressions, skin texture and head accessories to the training data images. FaceGen provides the tools for adding these modifications to synthetic face renderings. Further experimentation with the network architecture, hyper parameters and regularisation techniques could improve the generalisation of the network and reduce the number of parameters. The decoder part of our network particularly, which currently only consists of transposed convolutional layers, could be optimised. Changing the number of layers or replacing them could lead to a performance boost.

With respect to the creation of the synthetically rotated facial image, an obvious area of improvement would be to reduce the run-time of the procedure. A simple initial modification could be to change the model-fitting network from 3DDFA [18] to something that has a lower inference time. The work presented in [19] could be recruited to that end.

## Figures and Tables

**Table 1:** A List of all layers in the CNN implementation. The thin horizontal line separates the encoder and decoder network parts, but the actual network is not in any way split up.

Input	Layer	Kernel	Stride	Output
256 × 256 × 6	Convolution	3	2	128 × 128 × 32
128 × 128 × 32	Inverted Residual	3	-	64 × 64 × 96
64 × 64 × 96	Inverted Residual	3	-	32 × 32 × 144
32 × 32 × 144	Inverted Residual	3	-	16 × 16 × 192
16 × 16 × 192	Inverted Residual	3	-	8 × 8 × 576
8 × 8 × 576	Convolution	3	2	8 × 8 × 512
8 × 8 × 512	Transposed Convolution	4	1	8 × 8 × 512
8 × 8 × 512	Transposed Convolution	4	2	16 × 16 × 256
16 × 16 × 256	Transposed Convolution	4	1	16 × 16 × 256
16 × 16 × 256	Transposed Convolution	4	1	16 × 16 × 256
16 × 16 × 256	Transposed Convolution	4	2	32 × 32 × 128
32 × 32 × 128	Transposed Convolution	4	1	32 × 32 × 128
32 × 32 × 128	Transposed Convolution	4	1	32 × 32 × 128
32 × 32 × 128	Transposed Convolution	4	2	64 × 64 × 64
64 × 64 × 64	Transposed Convolution	4	1	64 × 64 × 64
64 × 64 × 64	Transposed Convolution	4	1	64 × 64 × 64
64 × 64 × 64	Transposed Convolution	4	2	128 × 128 × 32
128 × 128 × 32	Transposed Convolution	4	1	128 × 128 × 32
128 × 128 × 32	Transposed Convolution	4	2	256 × 256 × 16
256 × 256 × 16	Transposed Convolution	4	1	256 × 256 × 16
256 × 256 × 16	Transposed Convolution	4	1	256 × 256 × 3
256 × 256 × 3	Transposed Convolution	4	1	256 × 256 × 3
256 × 256 × 3	Transposed Convolution	4	1	256 × 256 × 3

**Table 2:** The NME of our proposed, synthetically trained, 2-input network against state-of-the-art. Best performance is highlighted in bold.

Methodology	Mean NME
3DDFA_V2 [19]	**0.0127**
PRN [4]	0.0134
Proposed 2-input Method	0.0164
VRN_Guided [16]	0.0187
3DDFA [18]	0.0227

**Table 3:** NME comparison for our transfer trained 2-input network and the current state-of-the-art. Best performance is highlighted in bold.

Methodology	Mean NME
Proposed 2-input Method	**0.0074**
3DDFA_2 [19]	0.0127
PRN [4]	0.0134
VRN_Guided [16]	0.0187
3DDFA [18]	0.0227

**Table 4:** Mean NME for various models. Best performance is highlighted in bold.

Methodology	Mean NME
Proposed 2-input Method	**0.0190**
3DDFA_V2 [19]	0.0193
PRN [4]	0.0204
VRN_Guided [16]	0.0286
Proposed 1-input Method	0.0310
3DDFA [18]	0.0346

## Data Availability

Publicly available datasets were analyzed in this study. The 300W-LP dataset can be found here: [http://www.cbsr.ia.ac.cn/users/xiangyuzhu/projects/3DDFA/main.htm] and the MICC Florence dataset can be found here: [https://www.micc.unifi.it/resources/datasets/florence-3d-faces/].
